# Supplementary figures and images for: GenDiS3 database: census on the prevalence of protein domain superfamilies of known structure in the entire sequence database
Source: Database (Oxford). 2025 May 9;2025:baaf035. doi: 10.1093/database/baaf035 (PMC12063530; doi:10.1093/database/baaf035)

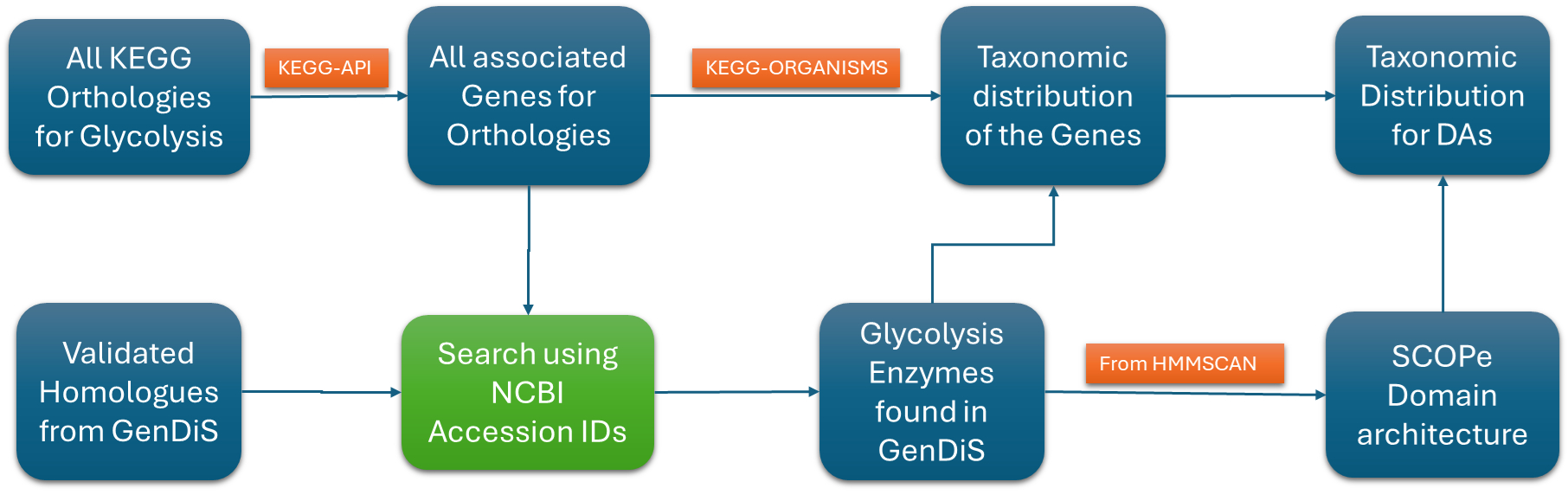

Supplement: baaf035_Supp [file baaf035_supp.zip › suppl_data/fig_s1.jpg]

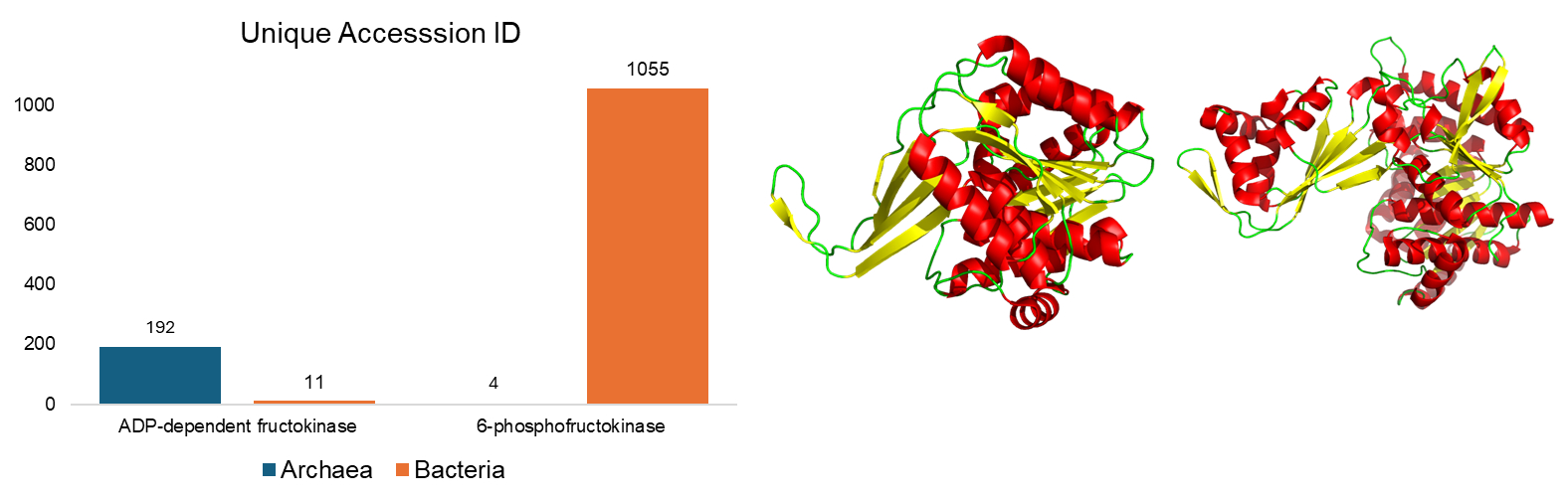

Supplement: baaf035_Supp [file baaf035_supp.zip › suppl_data/fig_s2.jpg]

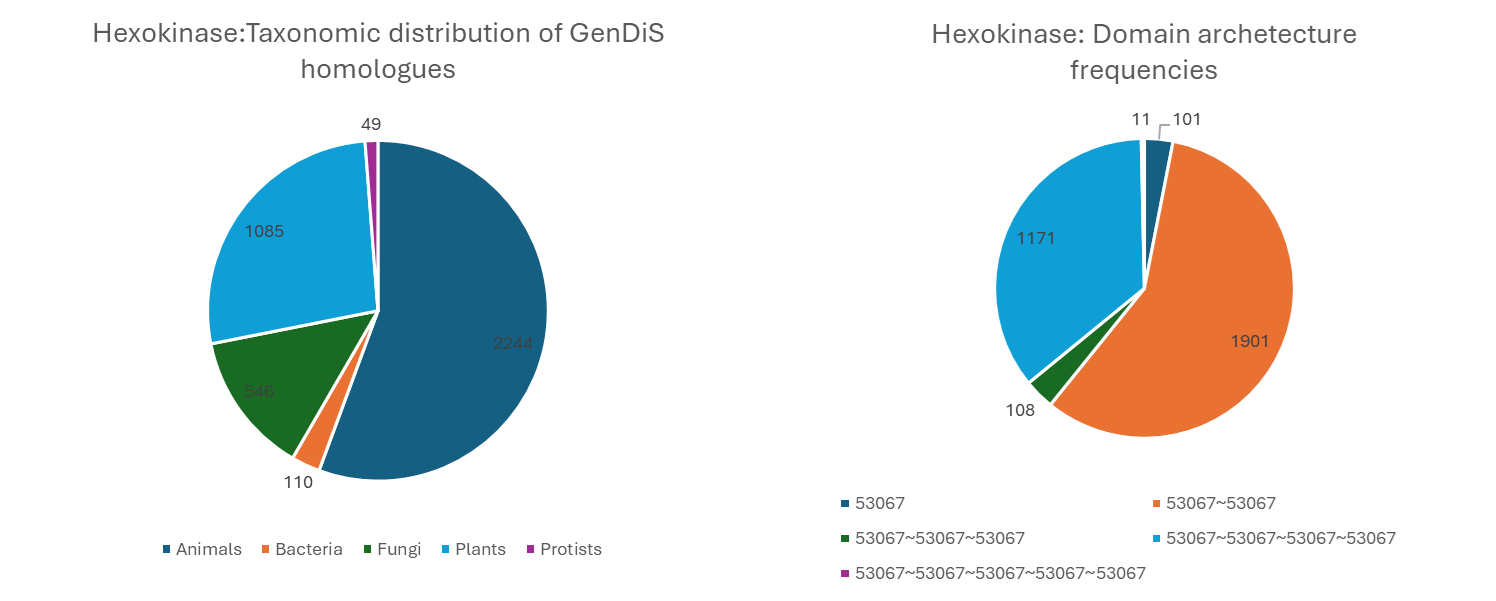

Supplement: baaf035_Supp [file baaf035_supp.zip › suppl_data/fig_s3.jpg]

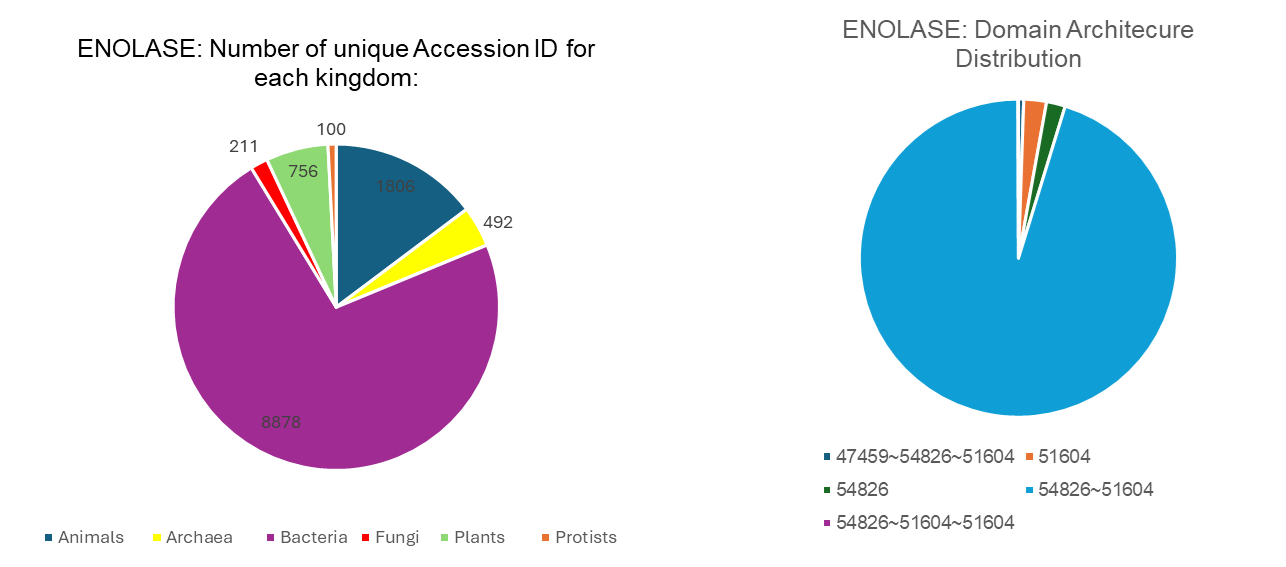

Supplement: baaf035_Supp [file baaf035_supp.zip › suppl_data/fig_s4.jpg]
